# The Role of Monocytes in Ischemic Stroke Pathobiology: New Avenues to Explore

**DOI:** 10.3389/fnagi.2016.00029

**Published:** 2016-02-22

**Authors:** Ayman ElAli, Noëmie Jean LeBlanc

**Affiliations:** ^1^Neuroscience Axis, CHU de Québec Research Center (CHUL)Québec City, QC, Canada; ^2^Department of Psychiatry and Neuroscience, Faculty of Medicine, Laval UniversityQuébec City, QC, Canada

**Keywords:** ischemic stroke, monocytes, inflammation, neuronal damage, neurorestoration

## Abstract

Ischemic stroke accounts for the majority of stroke cases and constitutes a major cause of death and disability in the industrialized world. Inflammation has been reported to constitute a major component of ischemic stroke pathobiology. In the acute phase of ischemic stroke, microglia, the resident macrophages of the brain, are activated, followed by several infiltration waves of different circulating immune cells into the brain. Among these circulating immune cells, monocytes have been shown to play a particularly important role. Following their infiltration, monocytes differentiate into potent phagocytic cells, the monocyte-derived macrophages (MDMs), in the ischemic brain. Initially, the presence of these cells was considered as marker of an exacerbated inflammatory response that contributes to brain damage. However, the recent reports are suggesting a more complex and multiphasic roles of these cells in ischemic stroke pathobiology. Monocytes constitute a heterogeneous group of cells, which comprises two major subsets in rodent and three major subsets in human. In both species, two equivalent subsets exist, the pro-inflammatory subset and the anti-inflammatory subset. Recent data have demonstrated that ischemic stroke differentially regulate monocyte subsets, which directly affect ischemic stroke pathobiology and may have direct implications in ischemic stroke therapies. Here, we review the recent findings that addressed the role of different monocyte subsets in ischemic stroke pathobiology, and the implications on therapies.

## Introduction

Stroke is the third leading cause of death and the first cause of disability in industrialized world. Ischemic stroke accounts for the majority of stroke cases, whereas the remaining stroke cases are hemorrhagic (Dirnagl et al., 1999). Regional blood supply disruption initiates the ischemic cascade that leads to neuronal death and rapid loss of neuronal function (Dirnagl et al., 1999). The ischemic cascade is characterized by the activation of several signaling pathways that compromise cell survival and function (Mehta et al., 2007). Ischemic stroke triggers blood-brain barrier (BBB) breakdown, thus contributing to the secondary progression of ischemic injury by increasing brain edema and exacerbating the inflammatory response in the sub-acute phase (hours to days after ischemic stroke onset; Dirnagl et al., 1999; Fagan et al., 2004). The severity of these early events reduces the capacity of neurons to recover in the chronic phase (days to weeks after ischemic stroke onset), thus significantly worsening stroke outcomes (Moskowitz et al., 2010).

Inflammation plays a central role in ischemic stroke pathobiology (Jin et al., 2010). Following ischemic stroke, microglia, which are brain resident macrophages, are activated and circulating immune cells, such as monocytes, neutrophils and lymphocytes are recruited to injury site (Jin et al., 2010). Among these immune cells, monocytes that give rise to macrophages play a particularly important role (Chiba and Umegaki, 2013). Initially, the presence of monocytes at the injury site has been suggested to contribute to ischemic injury exacerbation in the acute phase (minutes to hours after ischemic stroke onset; Chen et al., 2003). However, the experimental approaches that aimed at depleting these cells in ischemic stroke animal models worsened ischemic injury by destabilizing brain microvasculature (Gliem et al., 2012). These reports outline the complex and multifaceted role of monocytes in ischemic stroke pathobiology. As such, this mini-review aims to summarize and discuss the recent findings that addressed the role of different monocyte subsets in ischemic stroke pathobiology, which may have direct implication on stroke therapies.

## Monocytes

Monocytes, which arise from the hematopoietic system in the bone marrow (BM), play essential role in the innate immune responses (Thériault et al., 2015). Monocytes are incompletely differentiated cells (Geissmann et al., 2010), which have been shown to possess a highly phagocytic capacity and to elicit specific responses depending on the nature of stimuli within their microenvironment (Ginhoux and Jung, 2014). Therefore, to fulfill their role, monocytes are equipped with several sophisticated mechanisms that include scavenger receptors, low-density lipoprotein receptors, toll-like receptors, chemokine/cytokine receptors, Fcγ receptors and adhesions molecules (Ginhoux and Jung, 2014). Monocytes are characterized by the expression of several clusters of differentiation, such as CD115, CD11c, CD14 and CD16 in human or CD115, CD11b and Ly6C in rodent (Ginhoux and Jung, 2014). In parallel, both human and rodent monocytes express different levels of chemokine receptors, among which are the chemokine (C-X3-C motif) receptor 1 (CX3CR1) and chemokine (C-C motif) receptor 2 (CCR2; Auffray et al., 2009). In human, monocytes are regrouped in three main subsets based on their CD14 and CD16 expression levels, which are the classical subset (CD14^++^CD16^−^), the intermediate subset (CD14^++^CD16^+^) and the non-classical subset (CD14^+^CD16^++^; Naert and Rivest, 2013). In rodent, monocytes are regrouped into two main subsets based on chemokine receptor and Ly6C expression levels, namely the pro-inflammatory subset (CX3CR1^low^CCR2^+^Ly6C^high^) that has a short half-life and is actively recruited to inflamed tissues, contributing to the inflammatory response, and the anti-inflammatory subset (CX3CR1^high^CCR2^−^Ly6C^low^) that has a long half-life and is continuously patrolling the lumen of blood vessel, contributing to the maintenance of vascular homeostasis (Naert and Rivest, 2013). The specific interaction between each of monocyte subsets and different brain components has begin to emerge due to the technical advances that allow discriminating and specifically manipulating each of these subsets (Thériault et al., 2015). For instance, the mobilization of pro-inflammatory monocytes from the BM to blood circulation has been reported to be totally dependent of CCR2 signaling (Mildner et al., 2009). Upon mobilization, pro-inflammatory monocytes are capable of infiltrating the inflamed brain, where they differentiate into macrophages that are morphologically indistinguishable from resident microglial cells (Thériault et al., 2015). On the other hand, the survival of anti-inflammatory monocytes in the BM has been demonstrated to be dependent on the activity of the orphan nuclear receptor NR4A1 (Hanna et al., 2011). Interestingly, NR4A1 has been shown in parallel to be implicated in the differentiation of pro-inflammatory monocytes into anti-inflammatory monocytes in the blood circulation (Ginhoux and Jung, 2014). Upon differentiation, the anti-inflammatory monocytes have been shown to play an important role in surveying the vasculature and promoting vascular tissue repair and healing by eliminating cell debris and toxic elements (Michaud et al., 2013). In addition, some reports have suggested that the anti-inflammatory subset is implicated in replenishing resident perivascular macrophage population (Hanisch and Kettenmann, 2007).

## The Dynamic of Monocytes in Ischemic Stroke

### Monocytes in the Brain

Microglial cells are rapidly activated within minutes in the acute phase of ischemic stroke (Taylor and Sansing, 2013). Once activated, microglia can be polarized to adopt different phenotypes ranging between two extremes, the classically activated M1 phenotype that is involved in pro-inflammatory actions, and the alternatively activated M2 phenotype that is involved in anti-inflammatory actions and tissue healing (Prinz and Priller, 2014). Microglial activation peaks 2–3 days following ischemic onset and persists for several weeks (Denes et al., 2007). In contrast to microglial cell rapid response, monocyte-derived macrophages (MDMs) are recruited to the injured brain most abundantly 3–7 days following ischemic stroke, which coincide with the chronic phase of ischemic stroke (Breckwoldt et al., 2008). During the chronic phase of ischemic stroke adaptive endogenous restorative mechanisms are activated in the brain, which aim essentially at limiting ischemic damage expansion (Lo, 2008). These mechanisms include neuronal plasticity and remodeling, a process in which specialized phagocytic cells, such as microglia, seem to play an important role (ElAli and Rivest, 2015). Indeed, the specific ablation of microglial cells exacerbated post-ischemic stroke injury, thus outlining the role of these cells in the post-ischemic restorative process (Lalancette-Hébert et al., 2007). Importantly, the capacity of microglial cells to mediate these reparative mechanisms was hampered by the severity of ischemic injury (Denes et al., 2007). Microglial cells have been reported to be highly vulnerable to severe ischemic injury, which was shown to compromise cell cycle progression, and to induce a M1 pro-inflammatory phenotype (Ritzel et al., 2015). However, MDMs have been reported to be less vulnerable to severe ischemic injury and to be implicated essentially in the early clearance of cell debris within the damaged tissues 7 days following ischemic stroke onset (Ritzel et al., 2015). Taken together, these observations are highly important, as although MDMs are morphologically indistinguishable from resident microglial cells and their function overlaps with microglial cell function, these cells possess a higher phagocytic capacity (Malm et al., 2010), which may implicate a more efficacious role in the post-ischemic neuronal plasticity and remodeling processes. However, future studies are warranted in order to fully address this point.

These previous studies did not address the specific role of monocyte subsets. The emerging evidence is suggesting several functional differences between monocyte subsets in ischemic stroke. For instance, CCR2^+^ pro-inflammatory monocytes have been shown to early infiltrate the ischemic brain, where they differentiate into anti-inflammatory patrolling monocytes (Gliem et al., 2012). The selective depletion of CCR2^+^ pro-inflammatory monocytes infiltration into the injured brain, induced the hemorrhagic transformation of ischemic injury in mice subjected to transient middle cerebral artery occlusion (MCAo) due to the excessive bleeding caused by the rupture of the structurally fragile newly formed vessels within the infarct border zone (Gliem et al., 2012). These results highlight a previously unknown role of MDMs in maintaining brain microvasculature integrity. However the exact contribution of monocyte subsets is still elusive as it is not clear whether the differentiation of CCR2^+^ pro-inflammatory monocytes (Ly6C^high^) into anti-inflammatory monocytes (Ly6C^low^) is mandatory in this process. In line with these results, a recent study demonstrated that CCR2^+^ pro-inflammatory monocytes (Ly6C^high^) infiltration into the ischemic brain plays a beneficial role in limiting ischemic injury due to their subsequent differentiation into M2 anti-inflammatory phenotype MDMs and their capacity to promote the polarization of adjacent MDMs/microglia towards a M2 anti-inflammatory phenotype (Chu et al., 2015). Taken together, these results could provide new insights into the role of these cells in post-ischemic angiogenesis. Briefly, in rodent, minutes following ischemic stroke onset, several genes related to angiogenesis are upregulated in the brain, namely vascular endothelial growth factor (VEGF), which is accompanied by an increased formation of new vessels (Hermann and Zechariah, 2009). However, the biological significance of such a pro-angiogenic activity following ischemic stroke is still a matter of debate. Several hypotheses have been advanced, among which the “clean-up hypothesis” (Manoonkitiwongsa et al., 2001). This hypothesis proposes that newly formed vessels following ischemic stroke acts as tunnels that facilitate the infiltration of MDMs into the damaged region and the removal of necrotic cells within this region (Manoonkitiwongsa et al., 2001). For instance, the authors found out that the density of brain microvasculature increased only in the border of necrotic areas within the ischemic region, which was tightly associated to an increased number of MDMs (Manoonkitiwongsa et al., 2001). In contrast to the few studies that investigated the implication of CCR2^+^ pro-inflammatory (Ly6C^high^), the role of anti-inflammatory monocytes (Ly6C^low^) in ischemic stroke has never been investigated or addressed. However, one study has investigated the implication of anti-inflammatory monocytes (Ly6C^low^) specific depletion on brain injury following cerebral hypoxia-ischemia induced by Levine/Vannucci model in adult mice (Michaud et al., 2014). Anti-inflammatory monocytes (Ly6C^low^) depletion did not affect brain injury (Michaud et al., 2014). However, Levine/Vannucci model in adult mice does not constitute an optimal model to investigate ischemic stroke, as it is not clinically relevant in adults. In order to better translate the potential of preclinical experimental studies into clinics, the Stroke Therapy Academic Industry Roundtable (STAIR) provided several recommendations that highlighted the necessity of using focal/transient ischemic stroke models that generate a small infarct and an ischemic penumbra in order to better represent the clinical situation (Stroke Therapy Academic Industry Roundtable (STAIR), 1999; Gladstone et al., 2002). Therefore, future studies using clinically relevant ischemic stroke models in adult animals are warranted in order to fully address the specific role of anti-inflammatory monocytes (Ly6C^low^) in the ischemic brain. In one study, the time course of pro-inflammatory (Ly6C^high^) and anti-inflammatory (Ly6C^low^) infiltration into the ischemic brain of mice subjected to transient MCAo has been analyzed (Kim et al., 2014). Both monocyte subsets have significantly infiltrated the ischemic brain beginning of day 1 following ischemic stroke, peaked at day 3 and then declined at day 7 (Kim et al., 2014). Interestingly, the recruitment and infiltration of pro-inflammatory monocytes (Ly6C^high^) decreased over time, while the recruitment and infiltration of anti-inflammatory monocytes (Ly6C^low^) significantly increased (Kim et al., 2014). These results clearly highlight the presence of a dynamic shift in the recruitment and infiltration of monocyte subsets into the ischemic brain, or probably the differentiation of pro-inflammatory monocytes (Ly6C^high^) into anti-inflammatory monocytes (Ly6C^low^). As such, it conceivable to speculate, based on the function of each monocyte subset, that in the acute phase of ischemic pro-inflammatory monocytes (Ly6C^high^) are implicated in limiting ischemic stroke damage by eliminating necrotic cell debris and maintaining brain microvasculature stability, whereas in the sub-acute and chronic phases of ischemic stroke anti-inflammatory monocytes (Ly6C^low^) are implicated in tissue remodeling and healing.

### Monocytes in the Periphery

**Figure 1:**
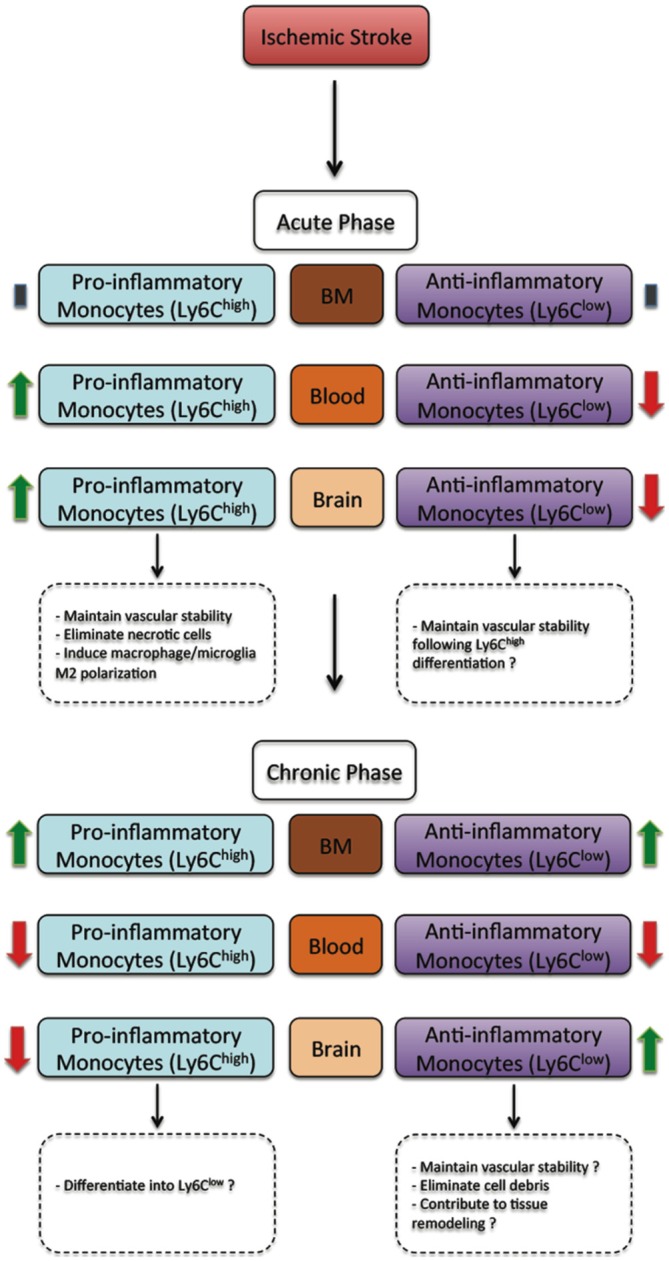
**The regulation of monocyte subsets following ischemic stroke in rodents.** In the acute phase, ischemic stroke does not affect monocyte subset number in the bone marrow (BM). However, the number of pro-inflammatory monocyte (Ly6C^high^) subset increases in blood circulation and the ischemic brain, whereas the number of anti-inflammatory monocyte (Ly6C^low^) subset decreases. In the acute phase, pro-inflammatory monocyte (Ly6C^high^) subset have been demonstrated to contribute to vascular stability, eliminating necrotic cells and promoting macrophage/microglia polarization towards a M2 protective phenotype, whereas anti-inflammatory monocyte (Ly6C^low^) subset may probably contribute to vascular stability. On the other hand, in the chronic phase, ischemic stroke increases the number of both pro-inflammatory monocyte (Ly6C^high^) and anti-inflammatory monocyte (Ly6C^low^) subsets in the BM. However, the number of both pro-inflammatory monocyte (Ly6C^high^) and anti-inflammatory monocyte (Ly6C^low^) subsets decreases. Finally, the number of pro-inflammatory monocyte (Ly6C^high^) subset decreases, whereas the number of anti-inflammatory monocyte (Ly6C^low^) subset increases. In the chronic phase, most infiltrated pro-inflammatory monocyte subset (Ly6C^high^) subset probably differentiates into anti-inflammatory monocyte (Ly6C^low^), thus contributing to vascular stability, eliminating cell debris and contributing to tissue remodeling and healing (Kim et al., 2014).

**Figure 2:**
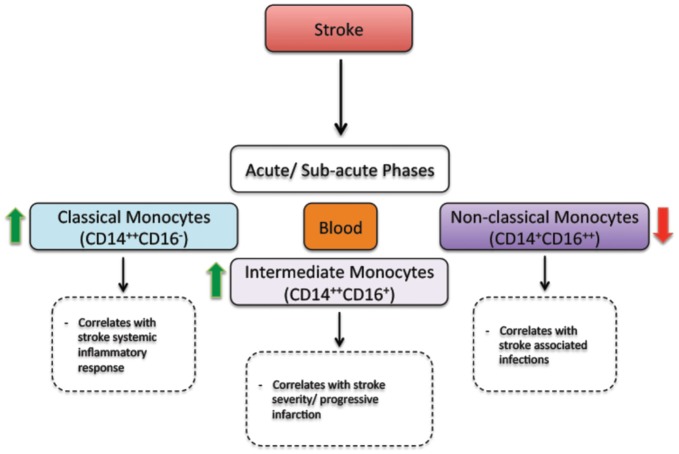
**The regulation of monocyte subsets following stroke in humans.** In the acute and sub-acute phases, stroke increases the number of classical monocytes (CD14^++^CD16^−^) and intermediate monocytes (CD14^++^CD16^+^) in blood circulation. On the other hand, stroke decreases the number of non-classical monocytes (CD14^+^CD16^++^) in blood circulation. Interestingly, the number of intermediate monocytes significantly increases in the blood circulation of patients presenting a sever injury (progressive infarction). In parallel, the number of non-classical monocytes decreases in the blood circulation of patients presenting stroke-associated infections (Kaito et al., 2013).

Besides triggering brain specific immune responses, ischemic stroke dynamically modulates the peripheral immune responses, which directly affect disease pathobiology and development (Dirnagl et al., 2007; Wang et al., 2007). In human, ischemic stroke triggered a sustained monocytosis translated by an increased number of total monocytes in the blood circulation of ischemic stroke patients (Kaito et al., 2013). Interestingly, the number of classical monocyte subset (CD14^++^CD16^−^), which is the equivalent of rodent pro-inflammatory monocyte subset (Ly6C^high^), significantly increased in blood circulation, whereas the number of the non-classical monocyte subset (CD14^+^CD16^++^), which is the equivalent of rodent anti-inflammatory monocyte subset (Ly6C^low^), significantly decreased (Kaito et al., 2013). This differential regulation of monocyte subsets in blood circulation of ischemic stroke patients, namely the increased number of the intermediate monocyte subset, and the decreased number of the non-classical monocyte subset in the acute and sub-acute phases of stroke, strongly correlated with the progression and severity of brain infarction (Kaito et al., 2013). Interestingly, a recent study showed that ischemic stroke, which was induced by MCAo, induces a temporal differential regulation of monocyte subsets in blood circulation and BM (Kim et al., 2014). More precisely, ischemic stroke potently increased the number of pro-inflammatory monocytes (Ly6C^high^) in blood circulation in the acute phase of ischemic stroke (3 h after ischemic stroke onset) and decreased their number below pre-ischemic levels in the sub-acute and chronic phases of ischemic stroke (from 1 to 7 days after ischemic stroke onset; Kim et al., 2014). On the other hand, ischemic stroke induced a sustained reduction of anti-inflammatory monocytes (Ly6C^low^) during the acute, sub-acute and chronic phases of ischemic stroke (Kim et al., 2014). Interestingly, the regulation patterns of monocyte subset are totally inversed in the BM. Importantly; spleen has been recently identified as an immediate reservoir of monocytes (Swirski et al., 2009). The number of the monocytes in the spleen is significantly higher than those present in blood circulation (Swirski et al., 2009). It has been previously shown that stroke induces spleen contraction, which was accompanied by a reduction in the number of splenic cells (Kim et al., 2014). Importantly, rat subjected to splenectomy 2 weeks before stroke, which was induced by permanent MCAo, attenuated brain injury and reduced the number of infiltrated macrophages into the ischemic brain, outlining the important of spleen-derived monocytes in stroke pathobiology (Ajmo et al., 2008). However, this study did not look into the implication of different monocyte subsets. In this regard, it has been recently shown that the number of pro-inflammatory (Ly6C^high^) and anti-inflammatory (Ly6C^low^) monocytes significantly decreased in the spleen of mice subjected to transient MCAo (Kim et al., 2014). Interestingly, the reduced number of monocyte subsets in the spleen strongly correlated with their increased number in the ischemic brain (Kim et al., 2014). Furthermore, the accumulation of pro-inflammatory (Ly6C^high^) and anti-inflammatory (Ly6C^low^) monocytes in the brain significantly decreased in mice subjected to splenectomy just before stroke induction. However, in this study monocytes reduced infiltration into the ischemic brain did not affect brain injury. Although authors concluded that spleen-derived monocytes have little implication in ischemic injury development in the acute phase, further studies are warranted in order to fully address the role of each subset by performing for example splenectomy in chimeric mice in which each monocyte subset is manipulated. Finally, the discrepancy between the above-discussed studies may be related to the specie and stroke model used (i.e., permanent vs. transient MCAo). Taking together, these observations, namely the recent experimental studies in rodents (Figure 1) and clinical investigations in stroke patients (Figure 2), outline the dynamic nature of ischemic-stroke induced regulation of monocyte subsets in different organs, which deserves further investigation to address its biological implication in ischemic stroke pathobiology.

## Conclusion

The overwhelming experimental data are suggesting that monocyte subsets and MDMs play complex and multiphasic roles in ischemic stroke pathobiology, displaying beneficial and adverse effects, which totally depend on injury severity and time window. These complex and multiphasic roles may explain the failure of most generalized anti-inflammatory therapeutic strategies that aim essentially at “switching-off” inflammation following ischemic stroke. The accumulating evidence is outlining a beneficial role of pro-inflammatory monocytes (Ly6C^high^) in the acute phase of ischemic stroke by limiting ischemic injury development, and pointing toward an important role of anti-inflammatory monocytes (Ly6C^low^) in post-ischemic brain remodeling. These new observations constitute a new framework to re-evaluate how global post-ischemic inflammation and more specifically monocyte subset differential and time/severity-dependent regulation are implicated in ischemic stroke pathobiology, which may allow the development of more adaptive and efficacious therapeutic strategies. In this regard, the overwhelming experimental findings are suggesting that strategies aiming at specifically modulating the number and/or activity of each monocyte subset at a specific time window may lead to the development of new therapeutic avenues new avenues in stroke therapies. For example, therapeutic strategies that aim at specifically boosting CCR2^+^ pro-inflammatory monocytes (Ly6C^high^) in the acute phase of ischemic stroke might limit brain injury progression and exacerbation. In parallel, therapeutic strategies that aim at specifically stimulating the activity of anti-inflammatory monocytes (Ly6C^low^) in the sub-acute and chronic phases might promote neurorestoration. Interestingly, clinical studies have sown that the increased number of intermediate monocytes (CD14^++^CD16^+^) in blood circulation of stroke patients is associated to brain injury severity, and the decreased number of non-classical monocytes (CD14^+^CD16^++^) is associated to post-stroke complications, namely stroke-associated infections. As such, it is conceivable to speculate that therapeutic strategies that aim at specifically reducing the number of intermediate monocytes (CD14^++^CD16^+^) in blood circulation in the acute phase of ischemic stroke, while increasing the number of non-classical monocytes (CD14^+^CD16^++^) might limit brain injury and reduce the risk of developing post-stroke complications. Indeed strategies that aim at modulating the function of monocyte subsets have been shown recently to be promising in treating neurodegenerative disorders, namely Alzheimer’s disease (AD), by promoting the elimination of vascular and parenchymal amyloid-beta (Aβ) toxic peptides (Michaud et al., 2013; ElAli et al., 2015). Therefore, the development of “immunomodulatory strategies” that specifically control the function of each monocyte subset at the different phases of ischemic stroke constitutes a promising approach in stroke therapies. However, to achieve this goal, further studies that aim at fully addressing the individual role of each monocyte subset following ischemic stroke are warranted. These studies should also assess the implication of stroke risk factors, by including human comorbid conditions such as hyperlipidemia, which has been shown to dynamically regulate and modulate the frequency, number and activity of circulating monocyte subsets (Mosig et al., 2009; Ley et al., 2011; Kim et al., 2012; Herz et al., 2014, 2015).

## Author Contributions

AEA planned, drafted and finalized the manuscript. NJL contributed in finalizing the manuscript.

## Conflict of Interest Statement

The authors declare that the research was conducted in the absence of any commercial or financial relationships that could be construed as a potential conflict of interest.
